# Supplementary figures and images for: Global transcriptome analysis of different stages of preimplantation embryo development in river buffalo
Source: PeerJ. 2019 Dec 2;7:e8185. doi: 10.7717/peerj.8185 (PMC6894430; doi:10.7717/peerj.8185)

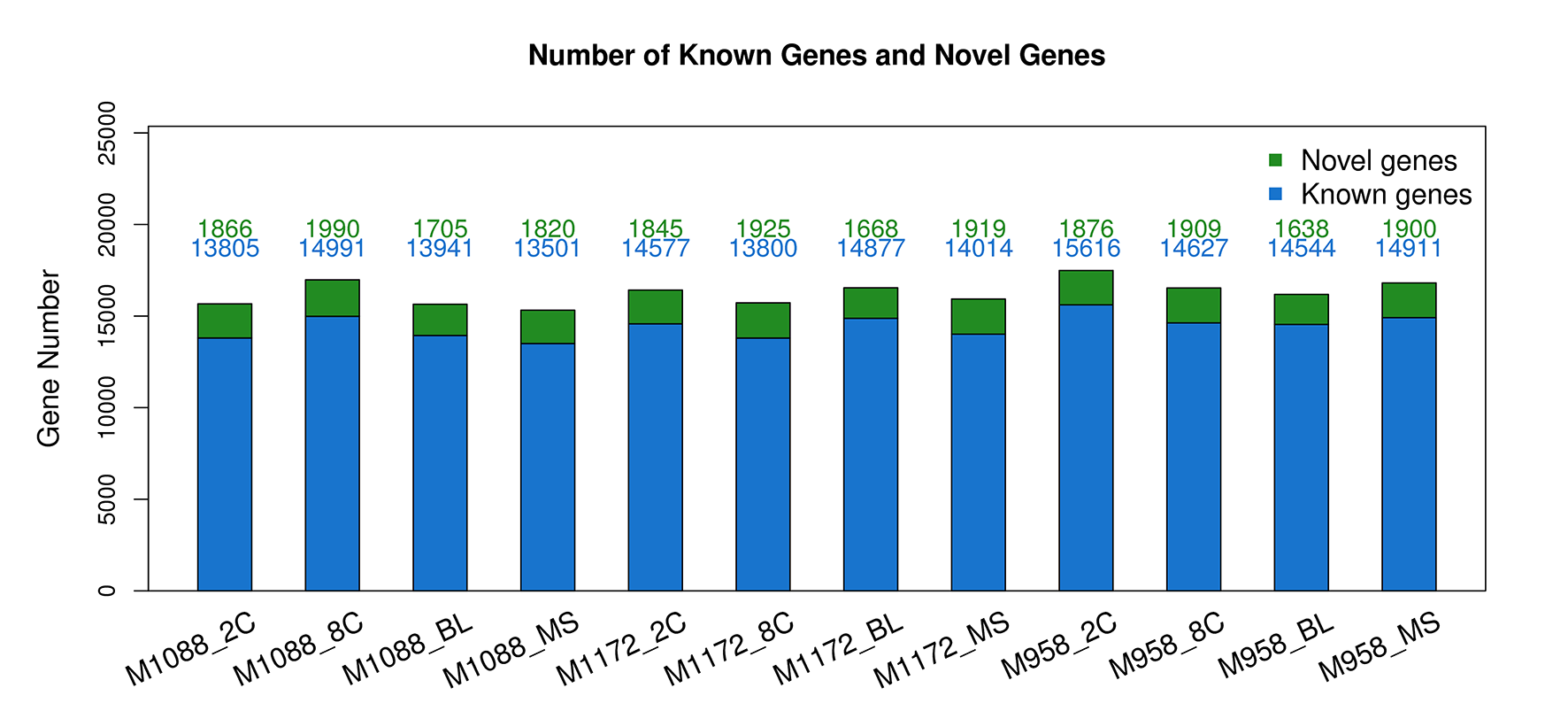

Supplement: Figure S1 [file peerj-07-8185-s001.png]

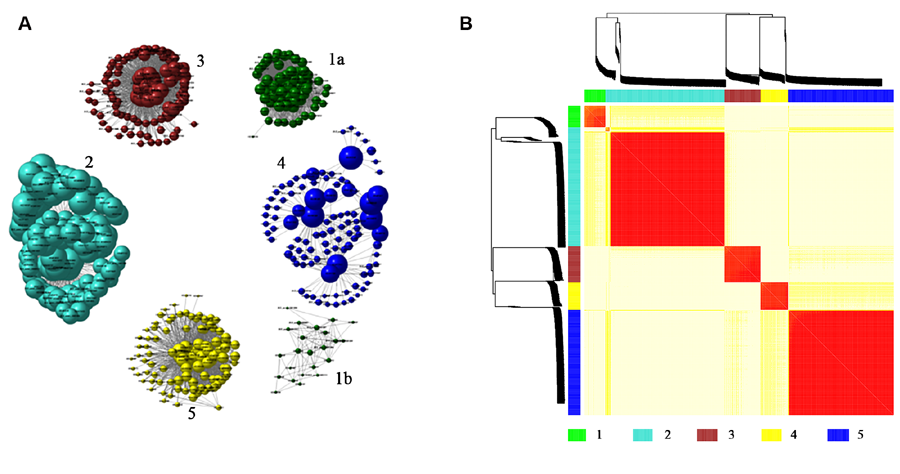

Supplement: Figure S2 — (A) Five modules of coexpression networks. (B) Corelation of the five modules. Deeper yellow coloring indicates stronger correlation. [file peerj-07-8185-s002.png]

# DEGs Number of The Most Enriched GOTerm

## 2C VS 8C

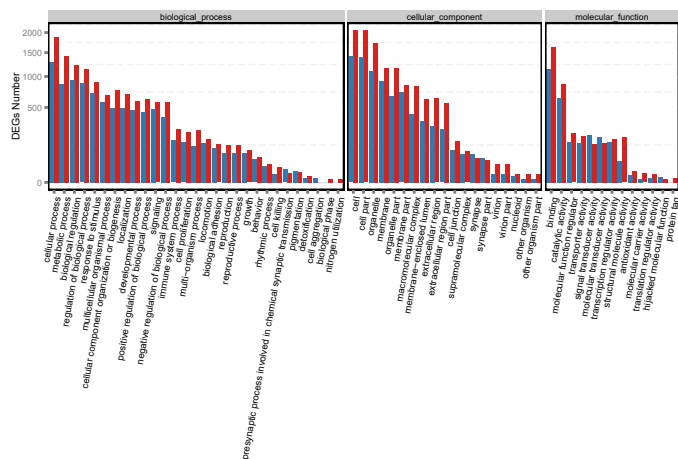

## 2C VS MS

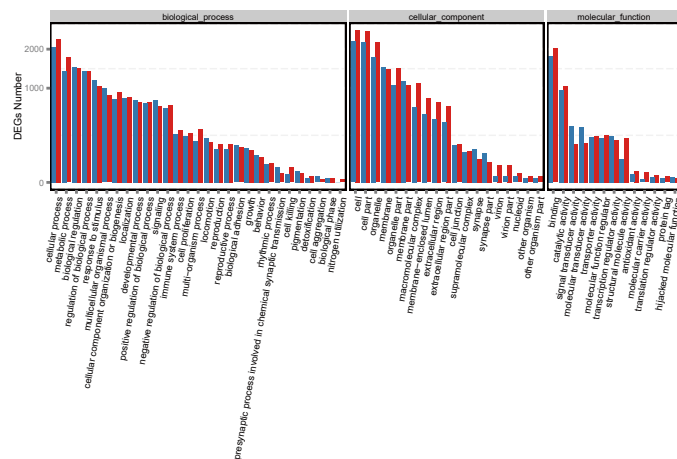

## 2C VS BL

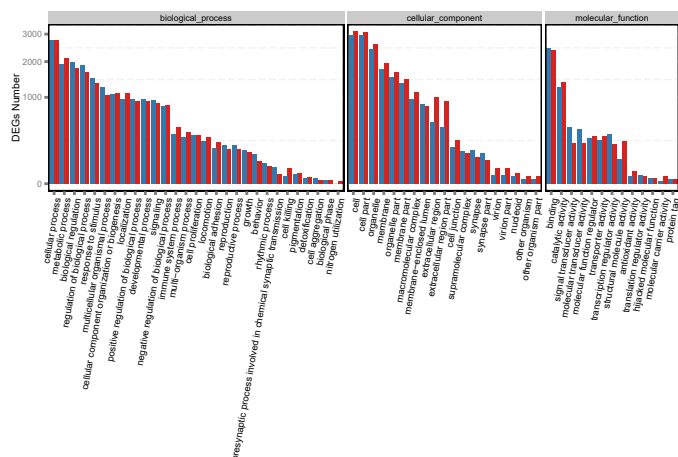

## 8C VS MS

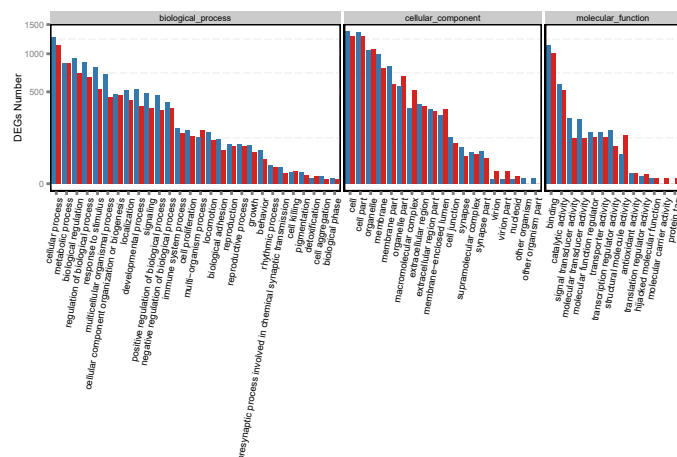

## 8C VS BL

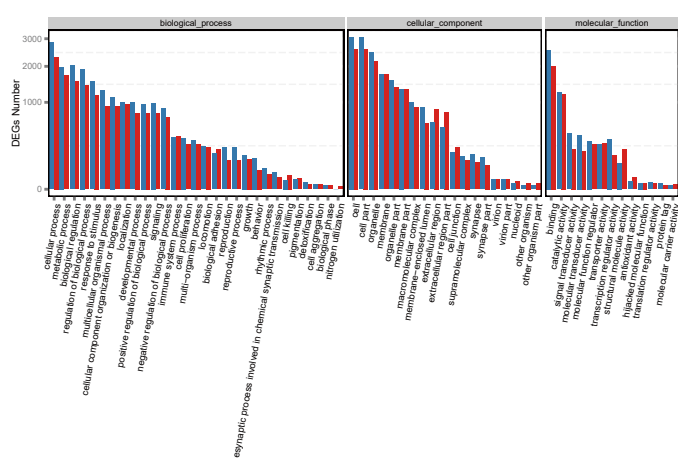

## MS VS BL

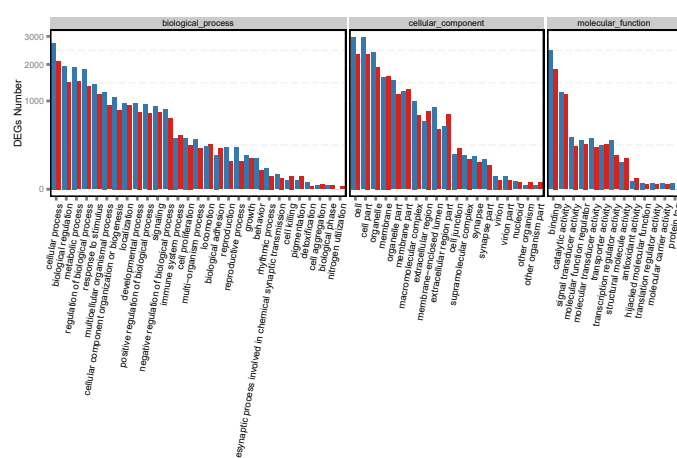

Supplement: Figure S4 — GO term annotation of DEGs for each comparison. [file peerj-07-8185-s004.pdf]

# DEGs Number of The Most Enriched Pathway

2C VS 8C

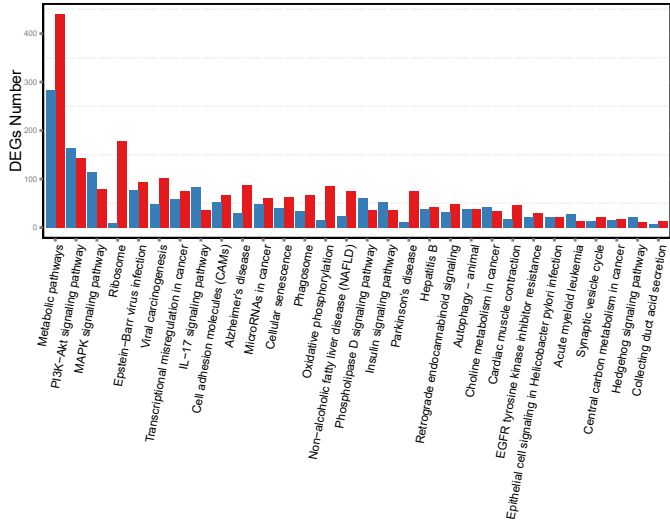

2C VS MS

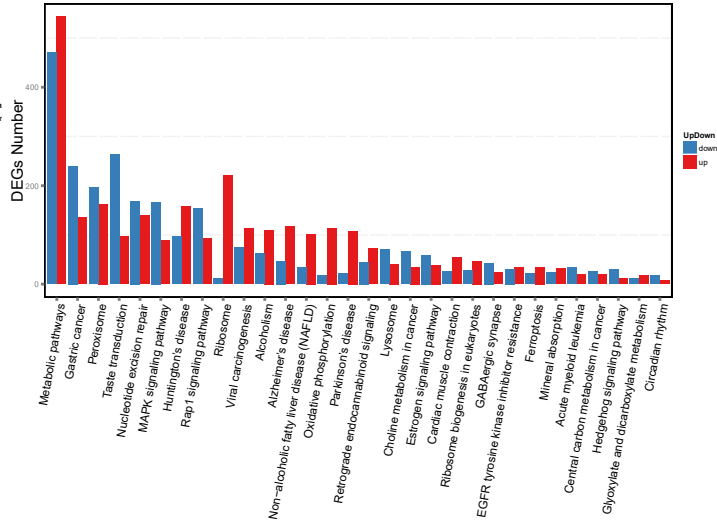

2C VS BL

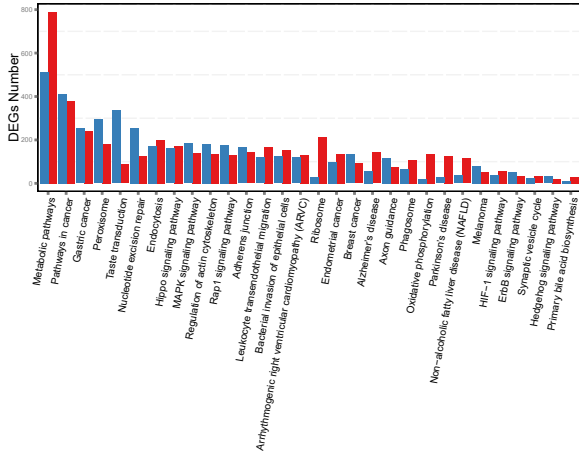

8C VS MS

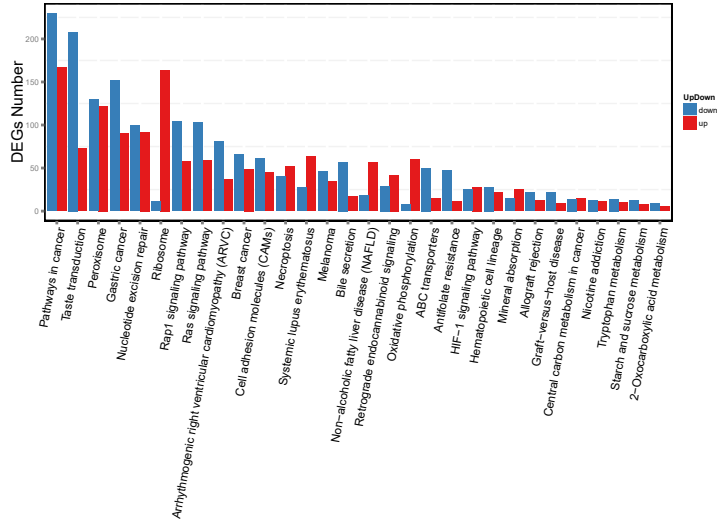

8C VS BL

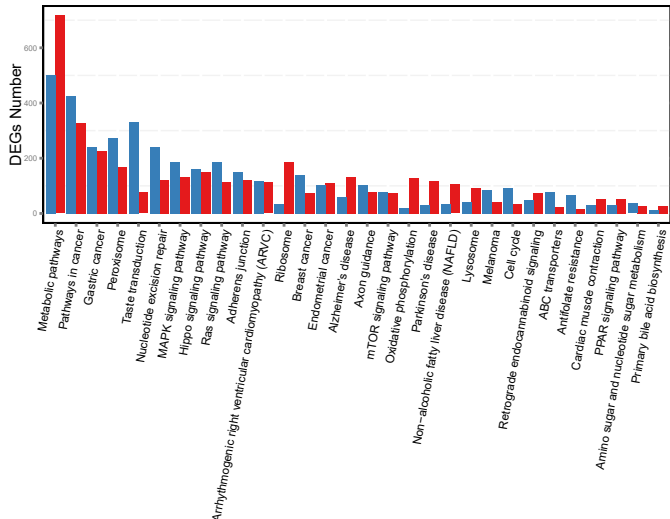

MS VS BL

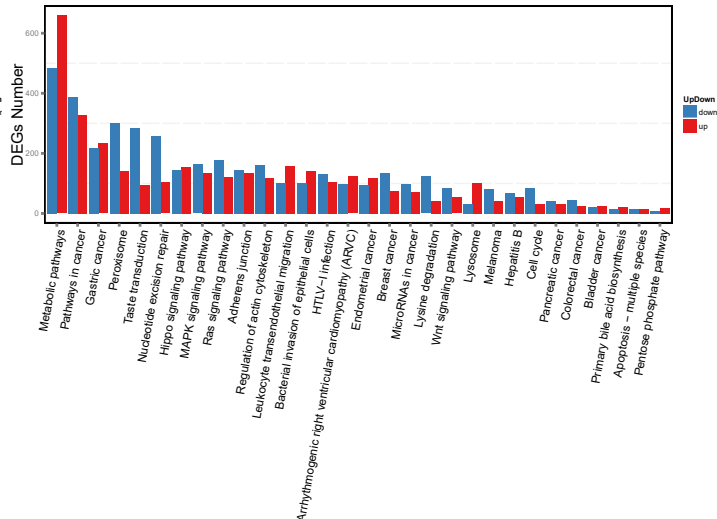

Supplement: Figure S5 — KEGG pathway annotation of DEGs. [file peerj-07-8185-s005.pdf]
