# Supplementary material for: Global transcriptome analysis of different stages of preimplantation embryo development in river buffalo
Source: PeerJ. 2019 Dec 2;7:e8185. doi: 10.7717/peerj.8185 (PMC6894430; doi:10.7717/peerj.8185)
Supplement: Figure S3 — GO analysis of coexpression modules. [file peerj-07-8185-s003.pdf]

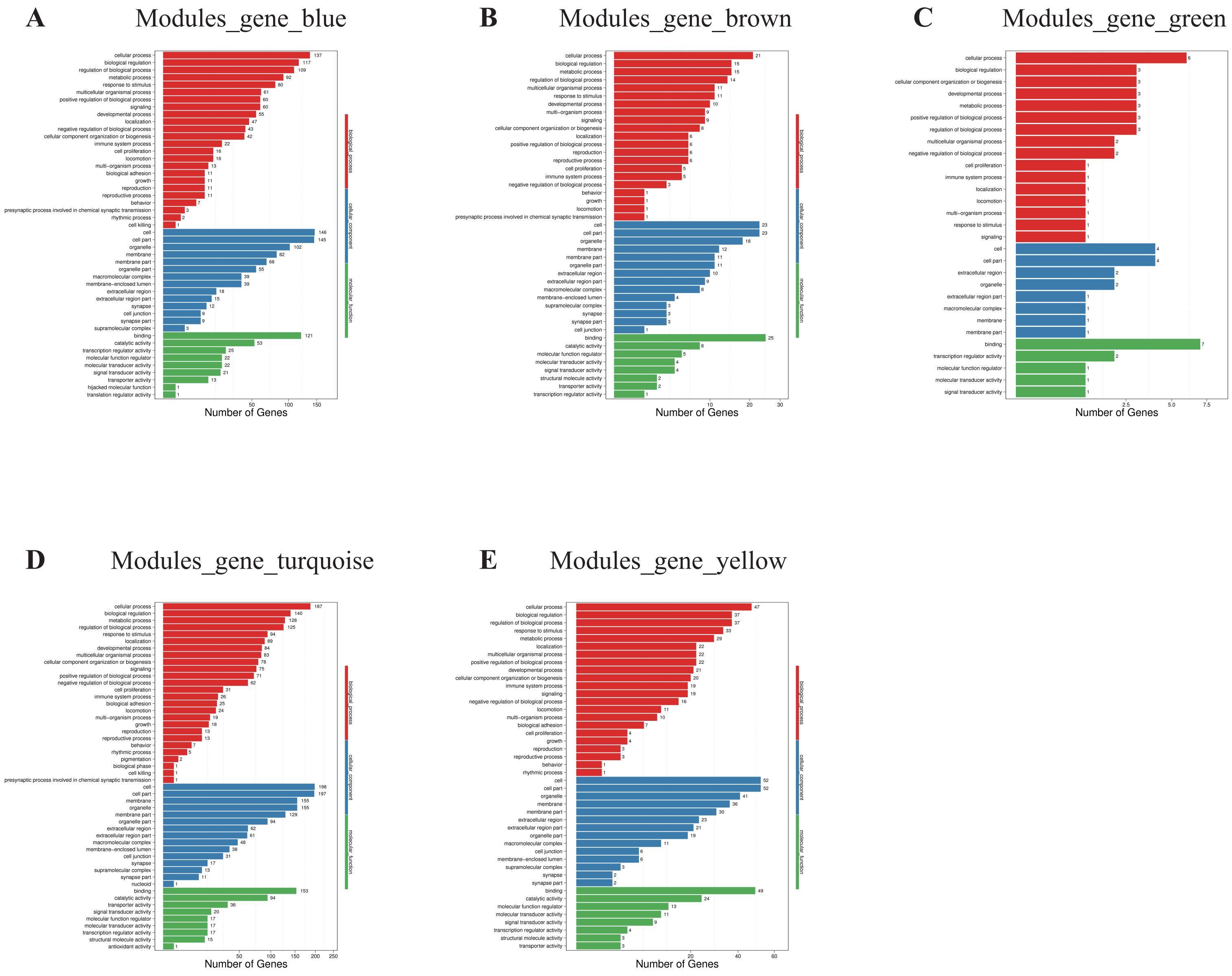
A
Modules_gene_blue
cellular process 137
biological regulation 117
regulation of biological process 109
metabolic process 92
response to stimulus 80
multicellular organismal process 61
positive regulation of biological process 60
signaling 60
developmental process 55
localization 47
negative regulation of biological process 43
cellular component organization or biogenesis 42
immune system process 22
cell proliferation 16
locomotion 16
multi−organism process 13
biological adhesion 11
growth 11
reproduction 11
reproductive process 11
behavior 7
presynaptic process involved in chemical synaptic transmission 3
rhythmic process 2
cell killing 1
cell 146
cell part 145
organelle 102
membrane 82
membrane part 68
organelle part 55
macromolecular complex 39
membrane−enclosed lumen 39
extracellular region 18
extracellular region part 15
synapse 12
cell junction 9
synapse part 9
supramolecular complex 3
binding 121
catalytic activity 53
transcription regulator activity 25
molecular function regulator 22
molecular transducer activity 22
signal transducer activity 21
transporter activity 13
hijacked molecular function 1
translation regulator activity 1
50 100 150
Number of Genes
biological process
cellular component
molecular function
B
Modules_gene_brown
cellular process 21
biological regulation 15
metabolic process 15
regulation of biological process 14
multicellular organismal process 11
response to stimulus 11
developmental process 10
multi−organism process 9
signaling 9
cellular component organization or biogenesis 8
localization 6
positive regulation of biological process 6
reproduction 6
reproductive process 6
cell proliferation 5
immune system process 5
negative regulation of biological process 3
behavior 1
growth 1
locomotion 1
presynaptic process involved in chemical synaptic transmission 1
cell 23
cell part 23
organelle 18
membrane 12
membrane part 11
organelle part 11
extracellular region 10
extracellular region part 9
macromolecular complex 8
membrane−enclosed lumen 4
supramolecular complex 3
synapse 3
synapse part 3
cell junction 1
binding 25
catalytic activity 8
molecular function regulator 5
molecular transducer activity 4
signal transducer activity 4
structural molecule activity 2
transporter activity 2
transcription regulator activity 1
10 20 30
Number of Genes
biological process
cellular component
molecular function
C
Modules_gene_green
cellular process 6
biological regulation 3
cellular component organization or biogenesis 3
developmental process 3
metabolic process 3
positive regulation of biological process 3
regulation of biological process 3
multicellular organismal process 2
negative regulation of biological process 2
cell proliferation 1
immune system process 1
localization 1
locomotion 1
multi−organism process 1
response to stimulus 1
signaling 1
cell 4
cell part 4
extracellular region 2
organelle 2
extracellular region part 1
macromolecular complex 1
membrane 1
membrane part 1
binding 7
transcription regulator activity 2
molecular function regulator 1
molecular transducer activity 1
signal transducer activity 1
2.5 5.0 7.5
Number of Genes
biological process
cellular component
molecular function
D
Modules_gene_turquoise
cellular process 187
biological regulation 140
metabolic process 128
regulation of biological process 125
response to stimulus 94
localization 89
developmental process 84
multicellular organismal process 83
cellular component organization or biogenesis 78
signaling 75
positive regulation of biological process 71
negative regulation of biological process 62
cell proliferation 31
immune system process 26
biological adhesion 25
locomotion 24
multi−organism process 19
growth 18
reproduction 13
reproductive process 13
behavior 7
rhythmic process 5
pigmentation 2
biological phase 1
cell killing 1
presynaptic process involved in chemical synaptic transmission 1
cell 198
cell part 197
membrane 155
organelle 155
membrane part 129
organelle part 94
extracellular region 62
extracellular region part 61
macromolecular complex 48
membrane−enclosed lumen 38
cell junction 31
synapse 17
supramolecular complex 13
synapse part 11
nucleoid 1
binding 153
catalytic activity 94
transporter activity 36
signal transducer activity 20
molecular function regulator 17
molecular transducer activity 17
transcription regulator activity 17
structural molecule activity 15
antioxidant activity 1
50 100 150 200 250
Number of Genes
biological process
cellular component
molecular function
E
Modules_gene_yellow
cellular process 47
biological regulation 37
regulation of biological process 37
response to stimulus 33
metabolic process 29
localization 22
multicellular organismal process 22
positive regulation of biological process 22
developmental process 21
cellular component organization or biogenesis 20
immune system process 19
signaling 19
negative regulation of biological process 16
locomotion 11
multi−organism process 10
biological adhesion 7
cell proliferation 4
growth 4
reproduction 3
reproductive process 3
behavior 1
rhythmic process 1
cell 52
cell part 52
organelle 41
membrane 36
membrane part 30
extracellular region 23
extracellular region part 21
organelle part 19
macromolecular complex 11
cell junction 6
membrane−enclosed lumen 6
supramolecular complex 3
synapse 2
synapse part 2
binding 49
catalytic activity 24
molecular function regulator 13
molecular transducer activity 11
signal transducer activity 9
transcription regulator activity 4
structural molecule activity 3
transporter activity 3
20 40 60
Number of Genes
biological process
cellular component
molecular function
