# Supplementary material for: Global transcriptome analysis of different stages of preimplantation embryo development in river buffalo
Source: PeerJ. 2019 Dec 2;7:e8185. doi: 10.7717/peerj.8185 (PMC6894430; doi:10.7717/peerj.8185)
Supplement: Table S1 [file peerj-07-8185-s006.docx]

**Table S1** Statistic of genes and transcripts numbers in all samples

| **Sample** | **Total Gene Number** | **Known Gene Number** | **Novel Gene Number** | **Total Transcript Number** | **Known Transcript Number** | **Novel Transcript Number** |
| --- | --- | --- | --- | --- | --- | --- |
| M958_2C | 17492 | 15616 | 1876 | 42768 | 23283 | 19485 |
| M958_8C | 16536 | 14627 | 1909 | 41125 | 19733 | 21392 |
| M958_BL | 16182 | 14544 | 1638 | 38319 | 20895 | 17424 |
| M958_MS | 16811 | 14911 | 1900 | 41228 | 20334 | 20894 |
| M1088_2C | 15671 | 13805 | 1866 | 40207 | 19291 | 20916 |
| M1088_8C | 16981 | 14991 | 1990 | 43485 | 21374 | 22111 |
| M1088_BL | 15646 | 13941 | 1705 | 32777 | 14307 | 18470 |
| M1088_MS | 15321 | 13501 | 1820 | 37949 | 17511 | 20438 |
| M1172_2C | 16422 | 14577 | 1845 | 42635 | 21172 | 21463 |
| M1172_8C | 15725 | 13800 | 1925 | 38927 | 17845 | 21082 |
| M1172_BL | 16545 | 14877 | 1668 | 39908 | 20384 | 19524 |
| M1172_MS | 15933 | 14014 | 1919 | 39604 | 17881 | 21723 |
