# Supplementary material for: Global transcriptome analysis of different stages of preimplantation embryo development in river buffalo
Source: PeerJ. 2019 Dec 2;7:e8185. doi: 10.7717/peerj.8185 (PMC6894430; doi:10.7717/peerj.8185)
Supplement: Table S2 [file peerj-07-8185-s007.docx]

**Table S2** Co-expression modules and genes in each modules

| **Modules**  **Gene** | **Blue** | **Brown** | **Green** | **Turquoise** | **Yellow** |
| --- | --- | --- | --- | --- | --- |
|  | 102388915 | 102388915 | 102388915 | 102388915 | 102388987 |
|  | 102388917 | 102388917 | 102388917 | 102388917 | 102389122 |
|  | 102388921 | 102388921 | 102388921 | 102388921 | 102389341 |
|  | 102388950 | 102388950 | 102388950 | 102388950 | 102389343 |
|  | 102389016 | 102389016 | 102389016 | 102389016 | 102389732 |
|  | 102389031 | 102389031 | 102389031 | 102389031 | 102390350 |
|  | 102389065 | 102389065 | 102389065 | 102389065 | 102390530 |
|  | 102389208 | 102389208 | 102389208 | 102389208 | 102390863 |
|  | 102389279 | 102389279 | 102389279 | 102389279 | 102391049 |
|  | 102389349 | 102389349 | 102389349 | 102389349 | 102391119 |
|  | 102389477 | 102389477 | 102389477 | 102389477 | 102391469 |
|  | 102389671 | 102389671 | 102389671 | 102389671 | 102391506 |
|  | 102389675 | 102389675 | 102389675 | 102389675 | 102391642 |
|  | 102389802 | 102389802 | 102389802 | 102389802 | 102391905 |
|  | 102390168 | 102390168 | 102390168 | 102390168 | 102392034 |
|  | 102390268 | 102390268 | 102390268 | 102390268 | 102392465 |
|  | 102390351 | 102390351 | 102390351 | 102390351 | 102392614 |
|  | 102390476 | 102390476 | 102390476 | 102390476 | 102393325 |
|  | 102390490 | 102390490 | 102390490 | 102390490 | 102394558 |
|  | 102390621 | 102390621 | 102390621 | 102390621 | 102394880 |
|  | 102390688 | 102390688 | 102390688 | 102390688 | 102394896 |
|  | 102390699 | 102390699 | 102390699 | 102390699 | 102395641 |
|  | 102390759 | 102390759 | 102390759 | 102390759 | 102395745 |
|  | 102390793 | 102390793 | 102390793 | 102390793 | 102396146 |
|  | 102390883 | 102390883 | 102390883 | 102390883 | 102396194 |
|  | 102390924 | 102390924 | 102390924 | 102390924 | 102396356 |
|  | 102390998 | 102390998 | 102390998 | 102390998 | 102396504 |
|  | 102391143 | 102391143 | 102391143 | 102391143 | 102396526 |
|  | 102391306 | 102391306 | 102391306 | 102391306 | 102396563 |
|  | 102391395 | 102391395 | 102391395 | 102391395 | 102396744 |
|  | 102391410 | 102391410 | 102391410 | 102391410 | 102396935 |
|  | 102391429 | 102391429 | 102391429 | 102391429 | 102397035 |
|  | 102391557 | 102391557 | 102391557 | 102391557 | 102397083 |
|  | 102391565 | 102391565 | 102391565 | 102391565 | 102397554 |
|  | 102391610 | 102391610 | 102391610 | 102391610 | 102397568 |
|  | 102391787 | 102391787 | 102391787 | 102391787 | 102397694 |
|  | 102391788 | 102391788 | 102391788 | 102391788 | 102397787 |
|  | 102391855 | 102391855 | 102391855 | 102391855 | 102397918 |
|  | 102391902 | 102391902 | 102391902 | 102391902 | 102397920 |
|  | 102391941 | 102391941 | 102391941 | 102391941 | 102398185 |
|  | 102392121 | 102392121 | 102392121 | 102392121 | 102398194 |
|  | 102392149 | 102392149 | 102392149 | 102392149 | 102398200 |
|  | 102392245 | 102392245 | 102392245 | 102392245 | 102398281 |
|  | 102392276 | 102392276 | 102392276 | 102392276 | 102398456 |
|  | 102392329 | 102392329 | 102392329 | 102392329 | 102398505 |
|  | 102392330 | 102392330 | 102392330 | 102392330 | 102398508 |
|  | 102392437 | 102392437 | 102392437 | 102392437 | 102398746 |
|  | 102392579 | 102392579 | 102392579 | 102392579 | 102399073 |
|  | 102392608 | 102392608 | 102392608 | 102392608 | 102399257 |
|  | 102392719 | 102392719 | 102392719 | 102392719 | 102399384 |
|  | 102392899 | 102392899 | 102392899 | 102392899 | 102399426 |
|  | 102392907 | 102392907 | 102392907 | 102392907 | 102399833 |
|  | 102393180 | 102393180 | 102393180 | 102393180 | 102400037 |
|  | 102393214 | 102393214 | 102393214 | 102393214 | 102400085 |
|  | 102393259 | 102393259 | 102393259 | 102393259 | 102400120 |
|  | 102393288 | 102393288 | 102393288 | 102393288 | 102400212 |
|  | 102393343 | 102393343 | 102393343 | 102393343 | 102400267 |
|  | 102393363 | 102393363 | 102393363 | 102393363 | 102400900 |
|  | 102393371 | 102393371 | 102393371 | 102393371 | 102400919 |
|  | 102393522 | 102393522 | 102393522 | 102393522 | 102401840 |
|  | 102393530 | 102393530 | 102393530 | 102393530 | 102402998 |
|  | 102393660 | 102393660 | 102393660 | 102393660 | 102403043 |
|  | 102394123 | 102394123 | 102394123 | 102394123 | 102403267 |
|  | 102394195 | 102394195 | 102394195 | 102394195 | 102403520 |
|  | 102394216 | 102394216 | 102394216 | 102394216 | 102403577 |
|  | 102394361 | 102394361 | 102394361 | 102394361 | 102404126 |
|  | 102394537 | 102394537 | 102394537 | 102394537 | 102404477 |
|  | 102394595 | 102394595 | 102394595 | 102394595 | 102404540 |
|  | 102394653 | 102394653 | 102394653 | 102394653 | 102405595 |
|  | 102394663 | 102394663 | 102394663 | 102394663 | 102405926 |
|  | 102394740 | 102394740 | 102394740 | 102394740 | 102405988 |
|  | 102394743 | 102394743 | 102394743 | 102394743 | 102406791 |
|  | 102394870 | 102394870 | 102394870 | 102394870 | 102406968 |
|  | 102394876 | 102394876 | 102394876 | 102394876 | 102407141 |
|  | 102394892 | 102394892 | 102394892 | 102394892 | 102407536 |
|  | 102394910 | 102394910 | 102394910 | 102394910 | 102408004 |
|  | 102395041 | 102395041 | 102395041 | 102395041 | 102408311 |
|  | 102395344 | 102395344 | 102395344 | 102395344 | 102408966 |
|  | 102395446 | 102395446 | 102395446 | 102395446 | 102409611 |
|  | 102395614 | 102395614 | 102395614 | 102395614 | 102409727 |
|  | 102395747 | 102395747 | 102395747 | 102395747 | 102410062 |
|  | 102395985 | 102395985 | 102395985 | 102395985 | 102411089 |
|  | 102396139 | 102396139 | 102396139 | 102396139 | 102411942 |
|  | 102396160 | 102396160 | 102396160 | 102396160 | 102412388 |
|  | 102396310 | 102396310 | 102396310 | 102396310 | 102412535 |
|  | 102396342 | 102396342 | 102396342 | 102396342 | 102412544 |
|  | 102396349 | 102396349 | 102396349 | 102396349 | 102412687 |
|  | 102396398 | 102396398 | 102396398 | 102396398 | 102413033 |
|  | 102396459 | 102396459 | 102396459 | 102396459 | 102413403 |
|  | 102396636 | 102396636 | 102396636 | 102396636 | 102414053 |
|  | 102396669 | 102396669 | 102396669 | 102396669 | 102414123 |
|  | 102396787 | 102396787 | 102396787 | 102396787 | 102414263 |
|  | 102396878 | 102396878 | 102396878 | 102396878 | 102414341 |
|  | 102396987 | 102396987 | 102396987 | 102396987 | 102414528 |
|  | 102397084 | 102397084 | 102397084 | 102397084 | 102415065 |
|  | 102397271 | 102397271 | 102397271 | 102397271 | 102415171 |
|  | 102397296 | 102397296 | 102397296 | 102397296 | 102415789 |
|  | 102397329 | 102397329 | 102397329 | 102397329 | 102416020 |
|  | 102397415 | 102397415 | 102397415 | 102397415 | 102416165 |
|  | 102397501 | 102397501 | 102397501 | 102397501 | 102416221 |
|  | 102397648 | 102397648 | 102397648 | 102397648 | 102416661 |
|  | 102397667 | 102397667 | 102397667 | 102397667 | 112580414 |
|  | 102397720 | 102397720 | 102397720 | 102397720 | 112582281 |
|  | 102397764 | 102397764 | 102397764 | 102397764 | BGI_novel_G000523 |
|  | 102397847 | 102397847 | 102397847 | 102397847 | BGI_novel_G000686 |
|  | 102398053 | 102398053 | 102398053 | 102398053 | BGI_novel_G001885 |
|  | 102398102 | 102398102 | 102398102 | 102398102 |  |
|  | 102398138 | 102398138 | 102398138 | 102398138 |  |
|  | 102398208 | 102398208 | 102398208 | 102398208 |  |
|  | 102398210 | 102398210 | 102398210 | 102398210 |  |
|  | 102398368 | 102398368 | 102398368 | 102398368 |  |
|  | 102398528 | 102398528 | 102398528 | 102398528 |  |
|  | 102398551 | 102398551 | 102398551 | 102398551 |  |
|  | 102398592 | 102398592 | 102398592 | 102398592 |  |
|  | 102398616 | 102398616 | 102398616 | 102398616 |  |
|  | 102398690 | 102398690 | 102398690 | 102398690 |  |
|  | 102398717 | 102398717 | 102398717 | 102398717 |  |
|  | 102398811 | 102398811 | 102398811 | 102398811 |  |
|  | 102398885 | 102398885 | 102398885 | 102398885 |  |
|  | 102398911 | 102398911 | 102398911 | 102398911 |  |
|  | 102398919 | 102398919 | 102398919 | 102398919 |  |
|  | 102399069 | 102399069 | 102399069 | 102399069 |  |
|  | 102399070 | 102399070 | 102399070 | 102399070 |  |
|  | 102399083 | 102399083 | 102399083 | 102399083 |  |
|  | 102399101 | 102399101 | 102399101 | 102399101 |  |
|  | 102399149 | 102399149 | 102399149 | 102399149 |  |
|  | 102399374 | 102399374 | 102399374 | 102399374 |  |
|  | 102399383 | 102399383 | 102399383 | 102399383 |  |
|  | 102399407 | 102399407 | 102399407 | 102399407 |  |
|  | 102399480 | 102399480 | 102399480 | 102399480 |  |
|  | 102399491 | 102399491 | 102399491 | 102399491 |  |
|  | 102399605 | 102399605 | 102399605 | 102399605 |  |
|  | 102399744 | 102399744 | 102399744 | 102399744 |  |
|  | 102399777 | 102399777 | 102399777 | 102399777 |  |
|  | 102399780 | 102399780 | 102399780 | 102399780 |  |
|  | 102399863 | 102399863 | 102399863 | 102399863 |  |
|  | 102399965 | 102399965 | 102399965 | 102399965 |  |
|  | 102400023 | 102400023 | 102400023 | 102400023 |  |
|  | 102400168 | 102400168 | 102400168 | 102400168 |  |
|  | 102400234 | 102400234 | 102400234 | 102400234 |  |
|  | 102400272 | 102400272 | 102400272 | 102400272 |  |
|  | 102400396 | 102400396 | 102400396 | 102400396 |  |
|  | 102400406 | 102400406 | 102400406 | 102400406 |  |
|  | 102400547 | 102400547 | 102400547 | 102400547 |  |
|  | 102400630 | 102400630 | 102400630 | 102400630 |  |
|  | 102400632 | 102400632 | 102400632 | 102400632 |  |
|  | 102400710 | 102400710 | 102400710 | 102400710 |  |
|  | 102400716 | 102400716 | 102400716 | 102400716 |  |
|  | 102400751 | 102400751 | 102400751 | 102400751 |  |
|  | 102400777 | 102400777 | 102400777 | 102400777 |  |
|  | 102400858 | 102400858 | 102400858 | 102400858 |  |
|  | 102400949 | 102400949 | 102400949 | 102400949 |  |
|  | 102400982 | 102400982 | 102400982 | 102400982 |  |
|  | 102401020 | 102401020 | 102401020 | 102401020 |  |
|  | 102401036 | 102401036 | 102401036 | 102401036 |  |
|  | 102401262 | 102401262 | 102401262 | 102401262 |  |
|  | 102401477 | 102401477 | 102401477 | 102401477 |  |
|  | 102401529 | 102401529 | 102401529 | 102401529 |  |
|  | 102401578 | 102401578 | 102401578 | 102401578 |  |
|  | 102401598 | 102401598 | 102401598 | 102401598 |  |
|  | 102401601 | 102401601 | 102401601 | 102401601 |  |
|  | 102401614 | 102401614 | 102401614 | 102401614 |  |
|  | 102401653 | 102401653 | 102401653 | 102401653 |  |
|  | 102401765 | 102401765 | 102401765 | 102401765 |  |
|  | 102401837 | 102401837 | 102401837 | 102401837 |  |
|  | 102401856 | 102401856 | 102401856 | 102401856 |  |
|  | 102401872 | 102401872 | 102401872 | 102401872 |  |
|  | 102401922 | 102401922 | 102401922 | 102401922 |  |
|  | 102401938 | 102401938 | 102401938 | 102401938 |  |
|  | 102402261 | 102402261 | 102402261 | 102402261 |  |
|  | 102402298 | 102402298 | 102402298 | 102402298 |  |
|  | 102402308 | 102402308 | 102402308 | 102402308 |  |
|  | 102402334 | 102402334 | 102402334 | 102402334 |  |
|  | 102402360 | 102402360 | 102402360 | 102402360 |  |
|  | 102402401 | 102402401 | 102402401 | 102402401 |  |
|  | 102402466 | 102402466 | 102402466 | 102402466 |  |
|  | 102402512 | 102402512 | 102402512 | 102402512 |  |
|  | 102402679 | 102402679 | 102402679 | 102402679 |  |
|  | 102402693 | 102402693 | 102402693 | 102402693 |  |
|  | 102402792 | 102402792 | 102402792 | 102402792 |  |
|  | 102402885 | 102402885 | 102402885 | 102402885 |  |
|  | 102403065 | 102403065 | 102403065 | 102403065 |  |
|  | 102403084 | 102403084 | 102403084 | 102403084 |  |
|  | 102403085 | 102403085 | 102403085 | 102403085 |  |
|  | 102403166 | 102403166 | 102403166 | 102403166 |  |
|  | 102403282 | 102403282 | 102403282 | 102403282 |  |
|  | 102403465 | 102403465 | 102403465 | 102403465 |  |
|  | 102403483 | 102403483 | 102403483 | 102403483 |  |
|  | 102403502 | 102403502 | 102403502 | 102403502 |  |
|  | 102403583 | 102403583 | 102403583 | 102403583 |  |
|  | 102403714 | 102403714 | 102403714 | 102403714 |  |
|  | 102403755 | 102403755 | 102403755 | 102403755 |  |
|  | 102403821 | 102403821 | 102403821 | 102403821 |  |
|  | 102403917 | 102403917 | 102403917 | 102403917 |  |
|  | 102403922 | 102403922 | 102403922 | 102403922 |  |
|  | 102404052 | 102404052 | 102404052 | 102404052 |  |
|  | 102404055 | 102404055 | 102404055 | 102404055 |  |
|  | 102404270 | 102404270 | 102404270 | 102404270 |  |
|  | 102404509 | 102404509 | 102404509 | 102404509 |  |
|  | 102404535 | 102404535 | 102404535 | 102404535 |  |
|  | 102404650 | 102404650 | 102404650 | 102404650 |  |
|  | 102404660 | 102404660 | 102404660 | 102404660 |  |
|  | 102404687 | 102404687 | 102404687 | 102404687 |  |
|  | 102404838 | 102404838 | 102404838 | 102404838 |  |
|  | 102404884 | 102404884 | 102404884 | 102404884 |  |
|  | 102404914 | 102404914 | 102404914 | 102404914 |  |
|  | 102404963 | 102404963 | 102404963 | 102404963 |  |
|  | 102404987 | 102404987 | 102404987 | 102404987 |  |
|  | 102405272 | 102405272 | 102405272 | 102405272 |  |
|  | 102405289 | 102405289 | 102405289 | 102405289 |  |
|  | 102405633 | 102405633 | 102405633 | 102405633 |  |
|  | 102405819 | 102405819 | 102405819 | 102405819 |  |
|  | 102405867 | 102405867 | 102405867 | 102405867 |  |
|  | 102405887 | 102405887 | 102405887 | 102405887 |  |
|  | 102405962 | 102405962 | 102405962 | 102405962 |  |
|  | 102406071 | 102406071 | 102406071 | 102406071 |  |
|  | 102406176 | 102406176 | 102406176 | 102406176 |  |
|  | 102406207 | 102406207 | 102406207 | 102406207 |  |
|  | 102406251 | 102406251 | 102406251 | 102406251 |  |
|  | 102406342 | 102406342 | 102406342 | 102406342 |  |
|  | 102406382 | 102406382 | 102406382 | 102406382 |  |
|  | 102406421 | 102406421 | 102406421 | 102406421 |  |
|  | 102406479 | 102406479 | 102406479 | 102406479 |  |
|  | 102406487 | 102406487 | 102406487 | 102406487 |  |
|  | 102406505 | 102406505 | 102406505 | 102406505 |  |
|  | 102406601 | 102406601 | 102406601 | 102406601 |  |
|  | 102406773 | 102406773 | 102406773 | 102406773 |  |
|  | 102406985 | 102406985 | 102406985 | 102406985 |  |
|  | 102407181 | 102407181 | 102407181 | 102407181 |  |
|  | 102407253 | 102407253 | 102407253 | 102407253 |  |
|  | 102407316 | 102407316 | 102407316 | 102407316 |  |
|  | 102407511 | 102407511 | 102407511 | 102407511 |  |
|  | 102407516 | 102407516 | 102407516 | 102407516 |  |
|  | 102407636 | 102407636 | 102407636 | 102407636 |  |
|  | 102407699 | 102407699 | 102407699 | 102407699 |  |
|  | 102407702 | 102407702 | 102407702 | 102407702 |  |
|  | 102407829 | 102407829 | 102407829 | 102407829 |  |
|  | 102407831 | 102407831 | 102407831 | 102407831 |  |
|  | 102407928 | 102407928 | 102407928 | 102407928 |  |
|  | 102407976 | 102407976 | 102407976 | 102407976 |  |
|  | 102407984 | 102407984 | 102407984 | 102407984 |  |
|  | 102408040 | 102408040 | 102408040 | 102408040 |  |
|  | 102408222 | 102408222 | 102408222 | 102408222 |  |
|  | 102408273 | 102408273 | 102408273 | 102408273 |  |
|  | 102408315 | 102408315 | 102408315 | 102408315 |  |
|  | 102408617 | 102408617 | 102408617 | 102408617 |  |
|  | 102408660 | 102408660 | 102408660 | 102408660 |  |
|  | 102408674 | 102408674 | 102408674 | 102408674 |  |
|  | 102408854 | 102408854 | 102408854 | 102408854 |  |
|  | 102408927 | 102408927 | 102408927 | 102408927 |  |
|  | 102409132 | 102409132 | 102409132 | 102409132 |  |
|  | 102409154 | 102409154 | 102409154 | 102409154 |  |
|  | 102409171 | 102409171 | 102409171 | 102409171 |  |
|  | 102409217 | 102409217 | 102409217 | 102409217 |  |
|  | 102409363 | 102409363 | 102409363 | 102409363 |  |
|  | 102409388 | 102409388 | 102409388 | 102409388 |  |
|  | 102409572 | 102409572 | 102409572 | 102409572 |  |
|  | 102409652 | 102409652 | 102409652 | 102409652 |  |
|  | 102409682 | 102409682 | 102409682 | 102409682 |  |
|  | 102409829 | 102409829 | 102409829 | 102409829 |  |
|  | 102409922 | 102409922 | 102409922 | 102409922 |  |
|  | 102410102 | 102410102 | 102410102 | 102410102 |  |
|  | 102410338 | 102410338 | 102410338 | 102410338 |  |
|  | 102410345 | 102410345 | 102410345 | 102410345 |  |
|  | 102410366 | 102410366 | 102410366 | 102410366 |  |
|  | 102410449 | 102410449 | 102410449 | 102410449 |  |
|  | 102410518 | 102410518 | 102410518 | 102410518 |  |
|  | 102410604 | 102410604 | 102410604 | 102410604 |  |
|  | 102410614 | 102410614 | 102410614 | 102410614 |  |
|  | 102410838 | 102410838 | 102410838 | 102410838 |  |
|  | 102410879 | 102410879 | 102410879 | 102410879 |  |
|  | 102410986 | 102410986 | 102410986 | 102410986 |  |
|  | 102411030 | 102411030 | 102411030 | 102411030 |  |
|  | 102411147 | 102411147 | 102411147 | 102411147 |  |
|  | 102411308 | 102411308 | 102411308 | 102411308 |  |
|  | 102411359 | 102411359 | 102411359 | 102411359 |  |
|  | 102411464 | 102411464 | 102411464 | 102411464 |  |
|  | 102411492 | 102411492 | 102411492 | 102411492 |  |
|  | 102411528 | 102411528 | 102411528 | 102411528 |  |
|  | 102411669 | 102411669 | 102411669 | 102411669 |  |
|  | 102411683 | 102411683 | 102411683 | 102411683 |  |
|  | 102411773 | 102411773 | 102411773 | 102411773 |  |
|  | 102411912 | 102411912 | 102411912 | 102411912 |  |
|  | 102411972 | 102411972 | 102411972 | 102411972 |  |
|  | 102412210 | 102412210 | 102412210 | 102412210 |  |
|  | 102412307 | 102412307 | 102412307 | 102412307 |  |
|  | 102412483 | 102412483 | 102412483 | 102412483 |  |
|  | 102412575 | 102412575 | 102412575 | 102412575 |  |
|  | 102412902 | 102412902 | 102412902 | 102412902 |  |
|  | 102412903 | 102412903 | 102412903 | 102412903 |  |
|  | 102412904 | 102412904 | 102412904 | 102412904 |  |
|  | 102412932 | 102412932 | 102412932 | 102412932 |  |
|  | 102413053 | 102413053 | 102413053 | 102413053 |  |
|  | 102413101 | 102413101 | 102413101 | 102413101 |  |
|  | 102413142 | 102413142 | 102413142 | 102413142 |  |
|  | 102413190 | 102413190 | 102413190 | 102413190 |  |
|  | 102413206 | 102413206 | 102413206 | 102413206 |  |
|  | 102413218 | 102413218 | 102413218 | 102413218 |  |
|  | 102413310 | 102413310 | 102413310 | 102413310 |  |
|  | 102413342 | 102413342 | 102413342 | 102413342 |  |
|  | 102413343 | 102413343 | 102413343 | 102413343 |  |
|  | 102413371 | 102413371 | 102413371 | 102413371 |  |
|  | 102413383 | 102413383 | 102413383 | 102413383 |  |
|  | 102413386 | 102413386 | 102413386 | 102413386 |  |
|  | 102413563 | 102413563 | 102413563 | 102413563 |  |
|  | 102413572 | 102413572 | 102413572 | 102413572 |  |
|  | 102413656 | 102413656 | 102413656 | 102413656 |  |
|  | 102413726 | 102413726 | 102413726 | 102413726 |  |
|  | 102413801 | 102413801 | 102413801 | 102413801 |  |
|  | 102413880 | 102413880 | 102413880 | 102413880 |  |
|  | 102413940 | 102413940 | 102413940 | 102413940 |  |
|  | 102413966 | 102413966 | 102413966 | 102413966 |  |
|  | 102414079 | 102414079 | 102414079 | 102414079 |  |
|  | 102414152 | 102414152 | 102414152 | 102414152 |  |
|  | 102414220 | 102414220 | 102414220 | 102414220 |  |
|  | 102414667 | 102414667 | 102414667 | 102414667 |  |
|  | 102414707 | 102414707 | 102414707 | 102414707 |  |
|  | 102414720 | 102414720 | 102414720 | 102414720 |  |
|  | 102414742 | 102414742 | 102414742 | 102414742 |  |
|  | 102414745 | 102414745 | 102414745 | 102414745 |  |
|  | 102414858 | 102414858 | 102414858 | 102414858 |  |
|  | 102414888 | 102414888 | 102414888 | 102414888 |  |
|  | 102415078 | 102415078 | 102415078 | 102415078 |  |
|  | 102415149 | 102415149 | 102415149 | 102415149 |  |
|  | 102415207 | 102415207 | 102415207 | 102415207 |  |
|  | 102415229 | 102415229 | 102415229 | 102415229 |  |
|  | 102415292 | 102415292 | 102415292 | 102415292 |  |
|  | 102415479 | 102415479 | 102415479 | 102415479 |  |
|  | 102415482 | 102415482 | 102415482 | 102415482 |  |
|  | 102415555 | 102415555 | 102415555 | 102415555 |  |
|  | 102415559 | 102415559 | 102415559 | 102415559 |  |
|  | 102415607 | 102415607 | 102415607 | 102415607 |  |
|  | 102415629 | 102415629 | 102415629 | 102415629 |  |
|  | 102415684 | 102415684 | 102415684 | 102415684 |  |
|  | 102416034 | 102416034 | 102416034 | 102416034 |  |
|  | 102416200 | 102416200 | 102416200 | 102416200 |  |
|  | 102416202 | 102416202 | 102416202 | 102416202 |  |
|  | 102416225 | 102416225 | 102416225 | 102416225 |  |
|  | 102416248 | 102416248 | 102416248 | 102416248 |  |
|  | 102416311 | 102416311 | 102416311 | 102416311 |  |
|  | 102416350 | 102416350 | 102416350 | 102416350 |  |
|  | 102416427 | 102416427 | 102416427 | 102416427 |  |
|  | 102416440 | 102416440 | 102416440 | 102416440 |  |
|  | 102416531 | 102416531 | 102416531 | 102416531 |  |
|  | 102416551 | 102416551 | 102416551 | 102416551 |  |
|  | 112577610 | 112577610 | 112577610 | 112577610 |  |
|  | 112577646 | 112577646 | 112577646 | 112577646 |  |
|  | 112577747 | 112577747 | 112577747 | 112577747 |  |
|  | 112578867 | 112578867 | 112578867 | 112578867 |  |
|  | 112582052 | 112582052 | 112582052 | 112582052 |  |
|  | 112582279 | 112582279 | 112582279 | 112582279 |  |
|  | 112582605 | 112582605 | 112582605 | 112582605 |  |
|  | 112583558 | 112583558 | 112583558 | 112583558 |  |
|  | 112584667 | 112584667 | 112584667 | 112584667 |  |
|  | 112585789 | 112585789 | 112585789 | 112585789 |  |
|  | BGI_novel_G000164 | BGI_novel_G000164 | BGI_novel_G000164 | BGI_novel_G000164 |  |
|  | BGI_novel_G000216 | BGI_novel_G000216 | BGI_novel_G000216 | BGI_novel_G000216 |  |
|  | BGI_novel_G000222 | BGI_novel_G000222 | BGI_novel_G000222 | BGI_novel_G000222 |  |
|  | BGI_novel_G000227 | BGI_novel_G000227 | BGI_novel_G000227 | BGI_novel_G000227 |  |
|  | BGI_novel_G000280 | BGI_novel_G000280 | BGI_novel_G000280 | BGI_novel_G000280 |  |
|  | BGI_novel_G000298 | BGI_novel_G000298 | BGI_novel_G000298 | BGI_novel_G000298 |  |
|  | BGI_novel_G000317 | BGI_novel_G000317 | BGI_novel_G000317 | BGI_novel_G000317 |  |
|  | BGI_novel_G000370 | BGI_novel_G000370 | BGI_novel_G000370 | BGI_novel_G000370 |  |
|  | BGI_novel_G000394 | BGI_novel_G000394 | BGI_novel_G000394 | BGI_novel_G000394 |  |
|  | BGI_novel_G000661 | BGI_novel_G000661 | BGI_novel_G000661 | BGI_novel_G000661 |  |
|  | BGI_novel_G000677 | BGI_novel_G000677 | BGI_novel_G000677 | BGI_novel_G000677 |  |
|  | BGI_novel_G000713 | BGI_novel_G000713 | BGI_novel_G000713 | BGI_novel_G000713 |  |
|  | BGI_novel_G000772 | BGI_novel_G000772 | BGI_novel_G000772 | BGI_novel_G000772 |  |
|  | BGI_novel_G000808 | BGI_novel_G000808 | BGI_novel_G000808 | BGI_novel_G000808 |  |
|  | BGI_novel_G000825 | BGI_novel_G000825 | BGI_novel_G000825 | BGI_novel_G000825 |  |
|  | BGI_novel_G000830 | BGI_novel_G000830 | BGI_novel_G000830 | BGI_novel_G000830 |  |
|  | BGI_novel_G000881 | BGI_novel_G000881 | BGI_novel_G000881 | BGI_novel_G000881 |  |
|  | BGI_novel_G000977 | BGI_novel_G000977 | BGI_novel_G000977 | BGI_novel_G000977 |  |
|  | BGI_novel_G001045 | BGI_novel_G001045 | BGI_novel_G001045 | BGI_novel_G001045 |  |
|  | BGI_novel_G001110 | BGI_novel_G001110 | BGI_novel_G001110 | BGI_novel_G001110 |  |
|  | BGI_novel_G001168 | BGI_novel_G001168 | BGI_novel_G001168 | BGI_novel_G001168 |  |
|  | BGI_novel_G001177 | BGI_novel_G001177 | BGI_novel_G001177 | BGI_novel_G001177 |  |
|  | BGI_novel_G001220 | BGI_novel_G001220 | BGI_novel_G001220 | BGI_novel_G001220 |  |
|  | BGI_novel_G001221 | BGI_novel_G001221 | BGI_novel_G001221 | BGI_novel_G001221 |  |
|  | BGI_novel_G001226 | BGI_novel_G001226 | BGI_novel_G001226 | BGI_novel_G001226 |  |
|  | BGI_novel_G001344 | BGI_novel_G001344 | BGI_novel_G001344 | BGI_novel_G001344 |  |
|  | BGI_novel_G001347 | BGI_novel_G001347 | BGI_novel_G001347 | BGI_novel_G001347 |  |
|  | BGI_novel_G001374 | BGI_novel_G001374 | BGI_novel_G001374 | BGI_novel_G001374 |  |
|  | BGI_novel_G001381 | BGI_novel_G001381 | BGI_novel_G001381 | BGI_novel_G001381 |  |
|  | BGI_novel_G001468 | BGI_novel_G001468 | BGI_novel_G001468 | BGI_novel_G001468 |  |
|  | BGI_novel_G001473 | BGI_novel_G001473 | BGI_novel_G001473 | BGI_novel_G001473 |  |
|  | BGI_novel_G001558 | BGI_novel_G001558 | BGI_novel_G001558 | BGI_novel_G001558 |  |
|  | BGI_novel_G001577 | BGI_novel_G001577 | BGI_novel_G001577 | BGI_novel_G001577 |  |
|  | BGI_novel_G001609 | BGI_novel_G001609 | BGI_novel_G001609 | BGI_novel_G001609 |  |
|  | BGI_novel_G001686 | BGI_novel_G001686 | BGI_novel_G001686 | BGI_novel_G001686 |  |
|  | BGI_novel_G001688 | BGI_novel_G001688 | BGI_novel_G001688 | BGI_novel_G001688 |  |
|  | BGI_novel_G001718 | BGI_novel_G001718 | BGI_novel_G001718 | BGI_novel_G001718 |  |
|  | BGI_novel_G001788 | BGI_novel_G001788 | BGI_novel_G001788 | BGI_novel_G001788 |  |
|  | BGI_novel_G001919 | BGI_novel_G001919 | BGI_novel_G001919 | BGI_novel_G001919 |  |
|  | BGI_novel_G001942 | BGI_novel_G001942 | BGI_novel_G001942 | BGI_novel_G001942 |  |
|  | BGI_novel_G001943 | BGI_novel_G001943 | BGI_novel_G001943 | BGI_novel_G001943 |  |
|  | BGI_novel_G002139 | BGI_novel_G002139 | BGI_novel_G002139 | BGI_novel_G002139 |  |
|  | BGI_novel_G002210 | BGI_novel_G002210 | BGI_novel_G002210 | BGI_novel_G002210 |  |
|  | BGI_novel_G002222 | BGI_novel_G002222 | BGI_novel_G002222 | BGI_novel_G002222 |  |
|  | BGI_novel_G002223 | BGI_novel_G002223 | BGI_novel_G002223 | BGI_novel_G002223 |  |
|  | BGI_novel_G002288 | BGI_novel_G002288 | BGI_novel_G002288 | BGI_novel_G002288 |  |
|  | BGI_novel_G002472 | BGI_novel_G002472 | BGI_novel_G002472 | BGI_novel_G002472 |  |
|  | BGI_novel_G002485 | BGI_novel_G002485 | BGI_novel_G002485 | BGI_novel_G002485 |  |
|  | BGI_novel_G002488 | BGI_novel_G002488 | BGI_novel_G002488 | BGI_novel_G002488 |  |
|  | BGI_novel_G002502 | BGI_novel_G002502 | BGI_novel_G002502 | BGI_novel_G002502 |  |
