# Supplementary material for: Global transcriptome analysis of different stages of preimplantation embryo development in river buffalo
Source: PeerJ. 2019 Dec 2;7:e8185. doi: 10.7717/peerj.8185 (PMC6894430; doi:10.7717/peerj.8185)
Supplement: Table S3 [file peerj-07-8185-s008.docx]

**Table S3** Formula of buffer in this study

| **BUFFER** | **FORMULA** |
| --- | --- |
| Storage buffer | NaCl, KCl, Na2HPO4, KH2PO4, CaCl2, MgCl2, Sodium pyruvate, Penicillin, Streptomycin, New-born calf serum, Heparinase |
| Culture medium | TCM199, NaHCO3, Sodium pyruvate, FSH, LH, FGF, E2, Cysteamine, Sodium lactate, Fetal cafl serum |
| Swimming up buffer | NaCl, KCl, Na2HPO4, KH2PO4, CaCl2, MgCl2, Sodium pyruvate, Penicillin, Streptomycin, Lysine, Phenol red, Hepes, BSA, Sodium lactate |
| Fertilization buffer | NaCl, KCl, Na2HPO4, KH2PO4, CaCl2, MgCl2, Sodium pyruvate, Penicillin, Streptomycin, Lysine, Phenol red, BSA, Sodium lactate, Heparinase |
| Embryo culture solution | TCM199, NaHCO3, Sodium pyruvate, Fetal cafl serum |
